# Exact Analytical Solution of the Peristaltic Nanofluids Flow in an Asymmetric Channel with Flexible Walls and Slip Condition: Application to the Cancer Treatment

**DOI:** 10.1155/2013/825376

**Published:** 2013-09-09

**Authors:** Abdelhalim Ebaid, Emad H. Aly

**Affiliations:** ^1^Department of Mathematics, Faculty of Science, University of Tabuk, Tabuk 71491, Saudi Arabia; ^2^Department of Mathematics, Faculty of Science, King Abdulaziz University, Jeddah 21589, Saudi Arabia; ^3^Department of Mathematics, Faculty of Education, Ain Shams University, Roxy, Cairo 11757, Egypt

## Abstract

In the cancer treatment, magnetic nanoparticles are injected into the blood vessel nearest to the cancer's tissues. The dynamic of these nanoparticles occurs under the action of the peristaltic waves generated on the flexible walls of the blood vessel. Studying such nanofluid flow under this action is therefore useful in treating tissues of the cancer. In this paper, the mathematical model describing the slip peristaltic flow of nanofluid was analytically investigated. Exact expressions were deduced for the temperature distribution and nano-particle concentration. In addition, the effects of the slip, thermophoresis, and Brownian motion parameters on the temperature and nano-particle concentration profiles were discussed and further compared with other approximate results in the literatures. In particular, these results have been obtained at the same values of the physical examined parameters that was considered in Akbar et al., “Peristaltic flow of a nanofluid with slip effects,” 2012. The results reveal that remarkable differences are detected between the exact current results and those approximately obtained in the literatures for behaviour of the temperature profile and nano-particles concentration. Accordingly, the current analysis and results are considered as optimal and therefore may be taken as a base for any future comparisons.

## 1. Introduction

In the recent times, peristalsis has attracted much attention due to its important engineering and medical applications, like chyme movement in the intestine, movement of eggs in the fallopian tube, transport of the spermatozoa in cervical canal, transport of bile in the bile duct, transport of cilia, circulation of blood in small blood vessels and in the intrauterine fluid flow within the uterine cavity. Since the first investigation of Latham [1], several theoretical and experimental studies have been conducted to understand peristaltic action [1–11]. In particular, to describe peristaltic flow in a symmetric channel or axisymmetric tubes containing Newtonian or nonNewtonian fluids, many models have been investigated by Zien and Ostrach [2], Lee and Fung [3], Srivastava et al. [4], El Shehawey and Mekheimer [6], Ramachandra and Usha [7], and Mekheimer and Abd elmaboud [10].

Further, present application is in the embryo transfer within the uterine cavity, where physiologists observed that the intra-uterine fluid flow due to myometrial contractions is peristaltic-type motion. In addition, De Vries et al. [12] found that the myometrial contractions may occur in both symmetric and asymmetric directions. Therefore, a great effort was devoted to study the peristaltic flow of Newtonian and nonNewtonian fluids in an asymmetric channel [13–23], and recently of Jeffrey and Johnson-Segalman fluids; see, for example, [24–28].

Although a huge number of studies for the peristaltic flow of classical fluids are available, only few papers are available for the peristaltic flow of nanofluids [29–34]. In this regard, Akbar et al. [33] may be the first authors to study the peristaltic nanofluids, in which the peristaltic flow and heat transfer of a nanofluid in an asymmetric channel have been analyzed. They have used the homotopy perturbation method to obtain the analytical approximate solutions for the temperature distribution and the nanoparticles concentration.

The nanofluids are a new class of fluids designed by dispersing nanometer-sized materials (nanoparticles, nanofibers, nanotubes, nanowires, nanorods, nanosheet, or droplets) in base fluids. Choi [35] reported that an innovative technique to improve heat transfer is by using nanoscale particles in the base fluid. Further, Choi et al. [36] showed that the addition of a small amount (less than 1% by volume) of nanoparticles to conventional heat transfer liquids increased the thermal conductivity of the fluid up to approximately two times.

In the tumors treatment, one of the effective methods is to inject the blood vessel nearest to the tumor with magnetic nanoparticles along with placing a magnet close to the tumor. These particles act like heat sources, in the presence of the applied magnetic field of alternating nature. Mekheimer and Abd elmaboud [10] found that the cancer's tissues are destroyed if the temperature reaches 42–45°C. On the other hand, in this application the drug may be placed on the magnetic nanoparticles and is injected near the tumor. Then, the drug is absorbed by the tumor through a high gradient magnetic field, which is concentrated near the tumor center [37]. Regarding, Habibi et al. [38] mentioned that the drug absorption due to high concentration of the magnetic particles increases and magnetic force prevents uniform drug distribution in circulatory system. This approach reduces the side effect and allows using high dose of anticancer drug. It should be noted that Majumder et al. [39] indicated that nanofluidic flow usually exhibits partial slip against the solid surface, which can be characterized by the so-called slip length, around 3.4–68 mm for different liquids.

The aim of this paper is to declare the exact effects of the slip, thermophoresis, and Brownian motion parameters on the temperature and nanoparticle concentration profiles of nanofluid flow in an asymmetric channel. It is well known that the exact solution of any physical model is optimal when available and would lead to the correct physical interpretations of the involved phenomena. Therefore, an approach is presented to achieve this goal for the resulting system of linear and nonlinear partial differential equations derived by Akbar et al. [33]. Then, these exact solutions are invested in obtaining the correct behaviour of the physical quantities. 

## 2. The Mathematical Investigated Model

**Figure 1:**
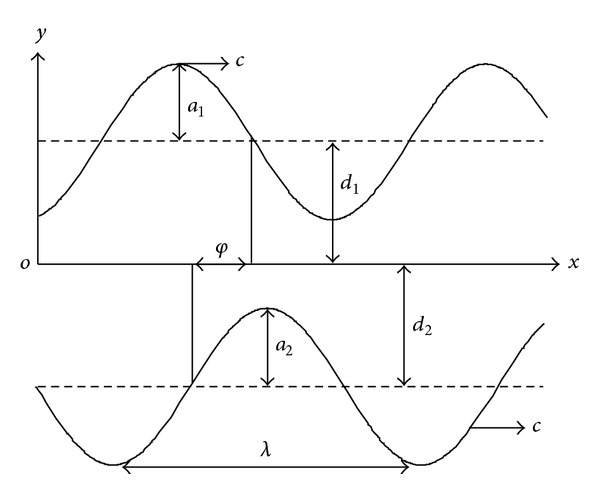
Schematic diagram of a two-dimensional asymmetric channel.

In the current work, we consider the peristaltic transport of an incompressible Newtonian nanofluid in an asymmetric channel with flexible walls, generating by propagation of waves on the channel walls traveling with different amplitudes and phases but with the same constant speed *c*. In the Cartesian coordinates system (*x*, *y*), the upper wall *h*_1_ and lower wall *h*_2_ are given by, see Figure 1,

(1) $$\begin{array}{c} h_{i}=Ad_{i}+a_{i}\cos\left[\frac{2\pi }{\lambda }\left(x-ct\right)+B\varphi \right], \end{array}$$

where *A* = 1, −1 and *B* = 0,1 when *i* = 1,2, respectively. Further, *a*_1_ and *a*_2_ are the amplitude of the waves, *λ* is the wave length, *d*_1_ + *d*_2_ is the width of the channel, the phase difference *φ* varies in the range 0 ≤ *φ* ≤ *π*, where *φ* = 0, and *π* corresponds to symmetric channel with waves out of the phase and in the phase, respectively. It should be noted that the following condition has to be achieved [40]:

(2) $$\begin{array}{c} a_{1}^{2}+a_{2}^{2}+2a_{1}a_{2}\cos\varphi \leq {\left(d_{1}+d_{2}\right)}^{2}, \end{array}$$

with the following nondimensional phenomena [33]:

(3) $$\begin{array}{c} a=\frac{a_{1}}{d_{1}},\;\;b=\frac{a_{2}}{d_{1}},\;\;d=\frac{d_{2}}{d_{1}}. \end{array}$$

On considering heat transfer along with nanoparticles phenomena under the assumptions of long wavelength and low Reynolds number approximation, Akbar et al. [33] found that the flow is governed by the following system of partial differential equations:

(4) $$\begin{array}{c} \psi_{yyyy}+G_{r}\theta_{y}+\beta_{r}\sigma_{y}=0, \end{array}$$

(5) $$\begin{array}{c} \theta_{yy}+N_{b}\theta_{y}\sigma_{y}+N_{t}{\left(\theta_{y}\right)}^{2}=0, \end{array}$$

(6) $$\begin{array}{c} \sigma_{yy}+\frac{N_{t}}{N_{b}}\theta_{yy}=0, \end{array}$$

(7) $$\begin{array}{c} \frac{dp}{dx}={\left(\psi_{yy}+G_{r}\theta +\beta_{r}\sigma \right)}_{y}, \end{array}$$

where *ψ*, *θ*, *σ*, and *p* are the stream function, temperature distribution, nanoparticles concentration, and pressure gradient, respectively. In addition, *N*_*b*_, *N*_*t*_, *G*_*r*_, and *B*_*r*_ are the Brownian motion parameter, thermophoresis parameter, local temperature Grashof number, and nanoparticles Grashof number, respectively. The system (4)–(6) has to be solved subject to the following boundary conditions on *ψ*, *θ*, and *σ*:

(8) $$\begin{array}{c} \psi =\frac{F}{2},\;\psi_{y}=-\beta \psi_{yy}-1\;\text{at}\;\;h_{1}=1+a\cos\left(x\right),\\ \psi =-\frac{F}{2},{\;\psi }_{y}=\beta \psi_{yy}-1\;\text{at}\;\;h_{2}=-d-b\cos\left(x+\varphi \right), \end{array}$$

(9) $$\begin{array}{c} \theta +\gamma \theta_{y}=0,\;\text{at}\;\;y=h_{1},\\ \theta -\gamma \theta_{y}=1,\;\text{at}\;\;y=h_{2},\\ \sigma -\gamma_{1}\sigma_{y}=1,\;\text{at}\;\;y=h_{2}. \end{array}$$

(10) $$\begin{array}{c} \sigma +\gamma_{1}\sigma_{y}=0,\;\text{at}\;\;y=h_{1},\\ \sigma -\gamma_{1}\sigma_{y}=1,\;\text{at}\;\;y=h_{2}. \end{array}$$

## 3. Closed Form Solution of the Model

In the present section, an effective procedure is introduced to obtain the analytical solutions for the resulted system of linear and nonlinear differential equations.

On integrating (6) twice and then inserting the resulted equation into (5), we obtain

(11) $$\begin{array}{c} \frac{\partial^{2}\theta }{\partial y^{2}}+N_{b}f_{1}\left(x\right)\frac{\partial \theta }{\partial y}=0. \end{array}$$

This equation can be exactly solved to give the temperature distribution, and therefore the nanoparticles concentration, as

(12) $$\begin{array}{c} \theta \left(x,y\right)=f_{4}\left(x\right)e^{-N_{b}f_{1}(x)y}+\frac{1}{N_{b}}\frac{f_{3}\left(x\right)}{f_{1}\left(x\right)}, \end{array}$$

(13) σ(x,y)=−NtNbf4(x)e−Nbf1(x)y +f1(x)y+f2(x)−NtNb2f3(x)f1(x),

where *f*_*i*_(*x*), *i* = 1, 2, 3, 4 are unknown functions to be determined. On applying the boundary conditions (9) on (12), and then solving the resulted equations, we get

(14) $$\begin{array}{c} f_{4}=\frac{1}{\left(1+\gamma N_{b}f_{1}\right)r_{2}^{f_{1}}-\left(1-\gamma N_{b}f_{1}\right)r_{1}^{f_{1}}},\\ f_{3}=\frac{-N_{b}f_{1}\left(1-\gamma N_{b}f_{1}\right)r_{1}^{f_{1}}}{\left(1+\gamma N_{b}f_{1}\right)r_{2}^{f_{1}}-\left(1-\gamma N_{b}f_{1}\right)r_{1}^{f_{1}}}. \end{array}$$

Further, applying the boundary conditions (10) on (13), and then solving the given system, results

(15) f2=NtNb2f3f1−(γ1f1−1Nb)Ntf4r1f1 −(γ1+h1)f1,

where

(16) $$\begin{array}{c} r_{1}=e^{-N_{b}h_{1}},{\;\;r}_{2}=e^{-N_{b}h_{2}}. \end{array}$$

The above analysis leads to the following implicit algebraic equation in *f*_1_(*x*):

(17) NtNb[(γ1Nbf1−1)r1f1+(γ1Nbf1+1)r2f1(γNbf1−1)r1f1+(γNbf1+1)r2f1]  +(2γ1+h1−h2)f1=−1.

## 4. Exact Solutions of the Physical Variables

### 4.1. Exact Expression of the Stream Function *ψ*(*x*, *y*)

Now, we search for the exact expression of the stream function *ψ*(*x*, *y*). By integrating the *ψ*(*x*, *y*) expression in (4) twice, we obtain

(18) $$\begin{array}{c} \psi =f_{8}+f_{7}y+\frac{1}{2}f_{6}y^{2}+\frac{1}{6}f_{5}y^{3}+g\left(y\right), \end{array}$$

where

(19) $$\begin{array}{c} \Omega_{1}\left(x\right)=\left(\frac{\beta_{r}N_{t}}{N_{b}}-G_{r}\right)\frac{1}{N_{b}}\frac{f_{3}}{f_{1}}-\beta_{r}f_{2},\\ \Omega_{2}\left(x\right)=\left(\frac{\beta_{r}N_{t}}{N_{b}}-G_{r}\right)f_{4},\\ g\left(y\right)=\frac{1}{6}\Omega_{1}y^{3}-\frac{1}{24}\beta_{r}f_{1}y^{4}-\frac{\Omega_{2}}{{\left(N_{b}f_{1}\right)}^{3}}e^{-N_{b}f_{1}y}. \end{array}$$

Applying the boundary conditions (8) on the *ψ*-equation given in (18) and (19), we obtain the following system:

(20) $$\begin{array}{c} f_{8}+f_{7}h_{i}+\frac{1}{2}f_{6}h_{i}^{2}+\frac{1}{6}f_{5}h_{i}^{3}=R_{i}\left(x\right),\\ f_{7}+A\left(\beta +h_{1}\right)f_{6}+\left(\frac{1}{2}h_{i}^{2}+A\beta h_{i}\right)f_{5}=S_{i}\left(x\right), \end{array}$$

where

(21) $$\begin{array}{c} R_{i}\left(x\right)=\frac{A}{2}F-g\left(h_{i}\right),\\ S_{i}\left(x\right)=-1-g^{\prime }\left(h_{i}\right)-A\beta g^{\prime \prime }\left(h_{i}\right), \end{array}$$

where again *A* = 1, −1 when *i* = 1, 2, respectively. 

#### 4.1.1. Obtaining the Values of *f*_*j*_, *j* = 5,…, 8

On solving the last linear system in (20) with (21), we obtain

(22)f5=6(−2R1+2R2+(h1−h2)(S1+S2))(h1−h2)2(6β+h1−h2),(23)  f6=2(−h13(S1+2S2)+3h12(R1−R2+(−2β+h2)S2)+3h1(2βR1−2βR2+h2((−2β+h2)S1+2βS2))−h2(3(−2β+h2)R1+(6β−3h2)R2+h2(2(−3β+h2)S1+h2S2)))×((h1−h2)2(2β+h1−h2)(6β+h1−h2))−1,f7=(h14S2+h13(2(−β+h2)S1+4βS2)+2h1(3(2β2−4βh2+h22)(R1−R2)+h22(−3β+h2)S2)+h2(6β(−2β+h2)R1+6β(2β−h2)R2+h22((−4β+h2)S1+2βS2))+h12(6(β−h2)R1−3(2(β−h2)R2 +h2((−2β+h2)S1+h2S2))))×((h1−h2)2(2β+h1−h2)(6β+h1−h2))−1,f8=h22(12β2−8βh2+h22)R1+h13(2β−h2)(4R2+h2(S1−2S2))+h14(R2−h2S2)+h12(12β2R2−6βh2(R1+3R2)+h23(2S1−S2)+3h22(R1+R2−2βS1+2βS2))−h1h2(2(6β2−9βh2+2h22)R1+6β(2β−h2)R2+h22((−4β+h2)S1+2βS2))×((h1−h2)2(2β+h1−h2)(6β+h1−h2))−1.

### 4.2. Exact Expression of the Pressure Gradient *dp*/*dx*

To get the pressure gradient *dp*/*dx*, we obtain from (7) and the above analysis that

(24) $$\begin{array}{c} \frac{dp}{dx}=\Omega_{3}\left(x\right)-\beta_{r}f_{1}y+\left(1+N_{b}f_{1}\right)\Omega_{2}\left(x\right)e^{-N_{b}f_{1}y}, \end{array}$$

where

(25) $$\begin{array}{c} \Omega_{3}\left(x\right)=\Omega_{1}\left(x\right)+f_{5}\left(x\right)+\beta_{r}f_{1}\left(x\right), \end{array}$$

and further all other functions are already well defined in the present section. 

### 4.3. Numerical Values of *f*_1_(*x*)

In Section 3, the general closed form solutions for the temperature distribution *θ* and nanoparticles concentration *σ* are obtained and expressed in terms of *f*_1_, *f*_2_, *f*_3_, and *f*_4_. As *f*_2_, *f*_3_, and *f*_4_ depend on the evaluating of *f*_1_, it is noticed from (17) that *f*_1_ is governed by a nonlinear algebraic equation. Once this equation is solved for *f*_1_, the analytical expressions for *θ* and *σ* are established.

It should be noted that obtaining the value of *f*_1_ analytically from (17) in terms of the other parameters set is a very difficult task, and it may be impossible. However, with the help of MATHEMATICA 6 software, the numerical solutions are still available. Values for *f*_1_ at some given cases are presented in Tables 1 and 2. These obtained values for *f*_1_ play an important role to get several plots for variation of the temperature distribution and nanoparticles concentration, which are introduced in the next section.

## 5. Results and Discussion

Besides discussing the effects of various physical parameters on the temperature distribution and nanoparticles concentration, comparing with the approximate solutions obtained by Akbar et al. [33] is also presented.

**Figure 2:**
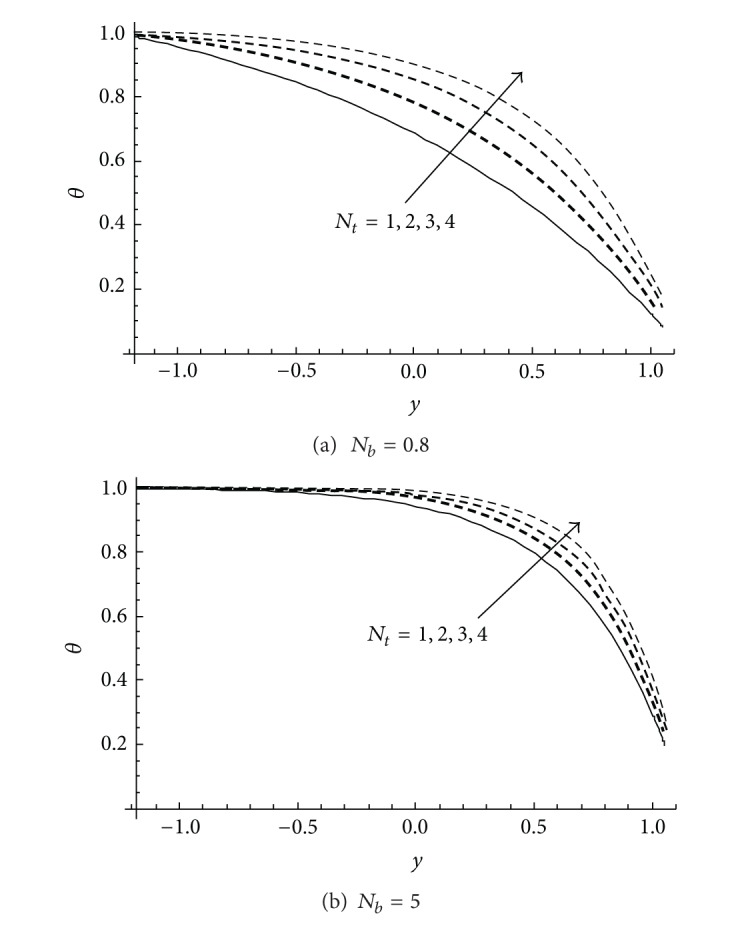
Variation of the temperature profile *θ* at different values of *N*_*t*_ for *a* = 0.1, *d* = 1, *b* = 0.5, *x* = 1, *ϕ* = 0.2 when *γ* = *γ*_1_ = 0.1 at (a) *N*_*b*_ = 0.8 and (b) *N*_*b*_ = 5.

Effect of *N*_*t*_ on the temperature profile *θ* for different values of the thermophoresis parameter *N*_*t*_ is plotted in Figure 2 at two different values for Brownian motion parameter *N*_*b*_. It is observed from this figure that the temperature profile increases when thermophoresis parameter *N*_*t*_ increases for the small or high value of Brownian motion parameter *N*_*b*_. It should be mentioned here that the present results are derived through exact solutions not as in [33] by an approximate way via the homotopy perturbation method. For the purpose of comparison, remarkable differences can be easily detected between our exact results presented in Figure 2(a) and those obtained by Akbar et al. [33] at the same values of the physical parameters. Regarding this we may point out that the approximate solutions obtained in [33] were not effective enough to give the correct physical curves.

**Figure 3:**
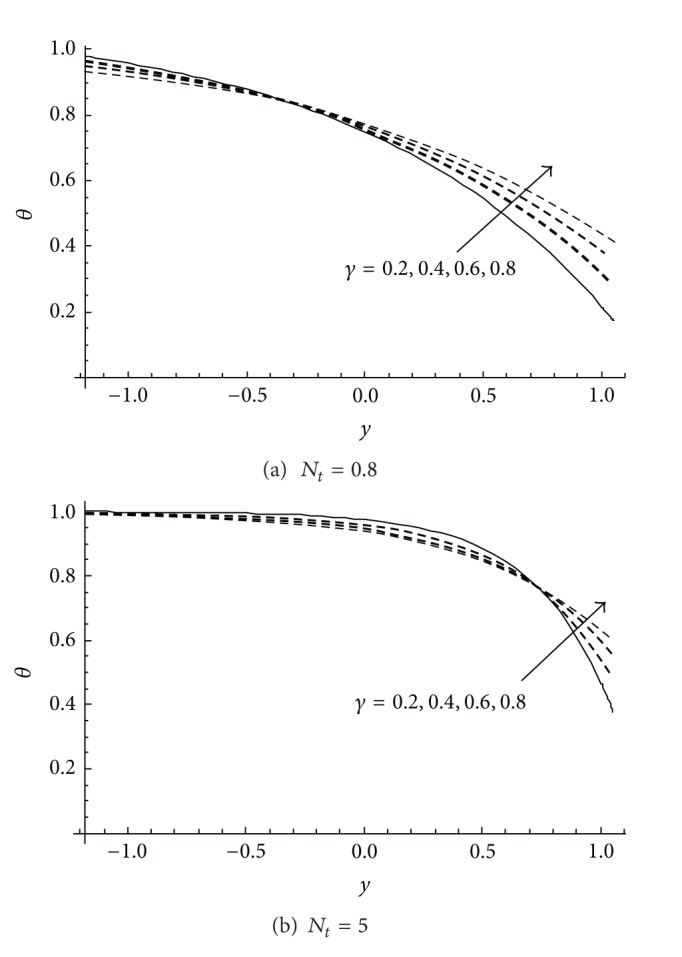
Variation of the temperature profile *θ* at different values of *γ* for *a* = 0.1, *d* = 1, *b* = 0.5, *x* = 1, *ϕ* = 0.2, *γ*_1_ = 0.5, *N*_*b*_ = 2 at (a) *N*_*t*_ = 0.8 and (b) *N*_*t*_ = 5.

Figure 3 shows the effect of the slip parameter *γ* on the temperature profile *θ* at two different values of the thermophoresis parameter *N*_*t*_. The results reveal that the temperature profile decreases in a specific domain with increasing *γ* for any small or high value of *N*_*t*_. After that domain, the behaviour of *θ* is different, where it increases with increasing *γ*. However, the domain in which the temperature profile decreases with increasing *γ* becomes wider when *N*_*t*_ takes high values; see Figure 3(b).

**Figure 4:**
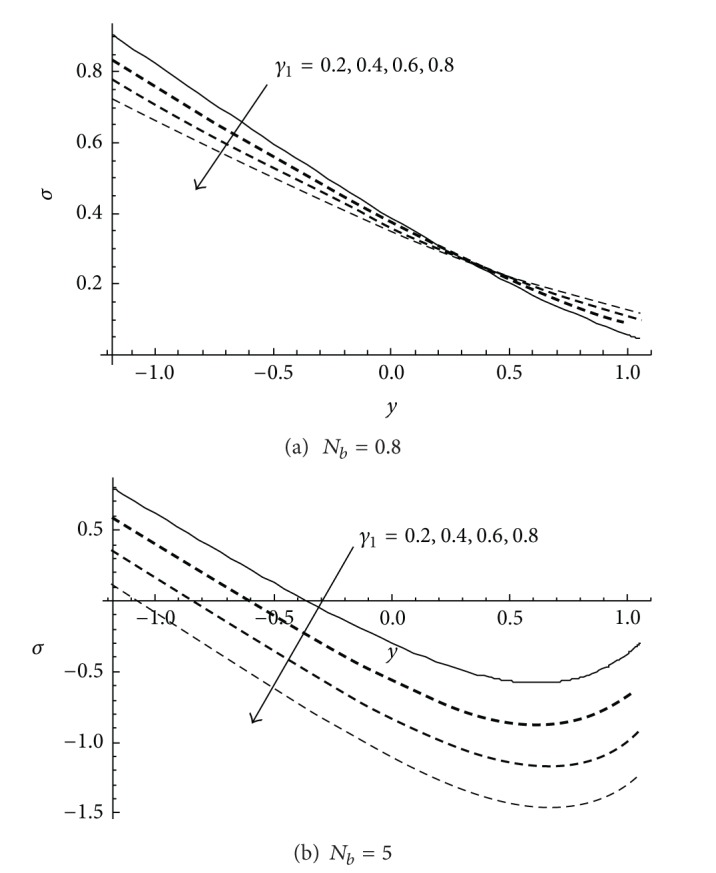
Variation profile of nanoparticles concentration *σ* at different values of *γ*_1_ for *a* = 0.1, *d* = 1, *b* = 0.5, *x* = 1, *ϕ* = 0.2, *γ* = 0.5, *N*_*b*_ = 2 at (a) *N*_*t*_ = 0.8 and (b) *N*_*t*_ = 5.

**Figure 5:**
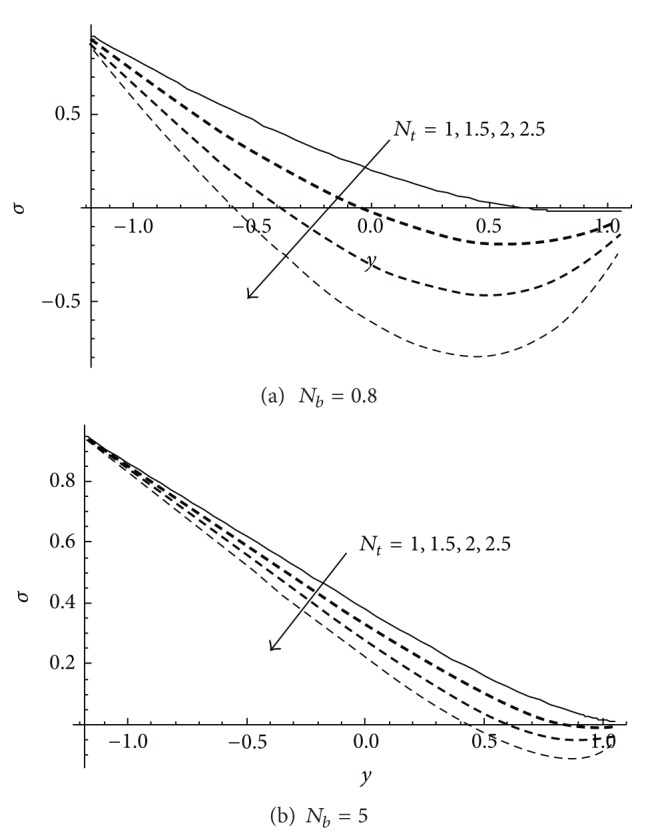
Variation profile of nanoparticles concentration *σ* at different values of *N*_*b*_ for *a* = 0.1, *d* = 1, *b* = 0.5, *x* = 1, *ϕ* = 0.2 when *γ* = *γ*_1_ = 0.1 at (a) *N*_*b*_ = 0.8 and (b) *N*_*b*_ = 5.

The nanoparticles concentration *σ* is depicted in Figures 4 and 5. In Figure 4(a) when the small value of Brownian motion parameter *N*_*b*_ is presented, it is observed that the nanoparticles concentration *σ* decreases in a certain domain with increasing the slip parameter *γ*_1_. A converse of this behaviour occurs after that domain. At a higher value of Brownian motion parameter *N*_*b*_, it is noticed that the nanoparticles concentration *σ* decreases in the whole domain with increasing the slip parameter *γ*_1_. On comparing the results depicted in Figure 4(a) and those obtained by Akbar et al. [33] at the same values of the physical parameters, slight differences are observed. Therefore, the current exact solutions, which can be verified by direct substitution into the governing differential equations and the boundary conditions, are reported in this paper for the first time. In addition, the effect of the thermophoresis parameter *N*_*t*_ on the nanoparticles concentration *σ* is depicted in Figure 5 at two different values for *N*_*b*_. The behaviour of *σ* always decreases with increasing *N*_*t*_. However, slight differences are also observed between the current results in Figure 5(b) and those obtained in Figure 4(b) by Akbar et al. [33]. Accordingly, the present results can be viewed as optimal and more accurate.

## 6. Conclusion

In this paper, exact effects of the slip conditions and peristaltic action on the nanofluid flow in an asymmetric channel were discussed for the variations of the temperature profile and nanoparticles concentration. The flow was described by a system of linear and nonlinear partial differential equations with complex boundary conditions generated on the flexible walls of the channel.

The exact solutions have been successfully obtained and reported for the first time. In addition, the obtained exact numerical results for effects of the slip condition, thermophoresis, and Brownian motion parameters on the temperature and nanoparticles concentration profiles show slight differences on comparing with the approximate solutions obtained via the homotopy perturbation method. The current analysis may throw some light on the nanofluid dynamic aspects used in the biomedical applications to treat the cancer's tissues, with the help of magnetic nanoparticles under the peristalsis on the blood vessels.

## Figures and Tables

**Table 1:** The numerical values of *f*_1_ at different values of *N*_*t*_ for *a* = 0.1, *d* = 1, *b* = 0.5, *x* = 1, *ϕ* = 0.2 when *γ* = *γ*_1_ = 0.1 for *N*_*b*_ = 0.8 and 5.

*N*_*b*_	*N*_*t*_	*f*_1_
0.8	1.0	−0.923945
	1.5	−1.180600
	2.0	−1.437250
	2.5	−1.693900
	3.0	−1.950550
	4.0	−2.463850

5.0	1.0	−0.492771
	1.5	−0.533835
	2.0	−0.574899
	2.5	−0.615964
	3.0	−0.657028
	4.0	−0.739156

**Table 2:** The numerical values of *f*_1_ at different values of *γ* and *γ*_1_ for *a* = 0.1, *d* = 1, *b* = 0.5, *x* = 1, *ϕ* = 0.2 when *N*_*b*_ = 2 for *N*_*t*_ = 0.8 and 5.

*N*_*t*_	*γ*_1_	*γ*	*f*_1_	*N*_*t*_	*γ*	*γ*_1_	*f*_1_
0.8	0.5	0.2	−0.468643	0.8	0.5	0.2	−0.496497
		0.4	−0.442588			0.4	−0.451474
		0.6	−0.424333			0.6	−0.415994
		0.8	−0.410694			0.8	−0.387347

5.0	0.5	0.2	−1.520890	5.0	0.5	0.2	−1.035780
		0.4	−1.176000			0.4	−1.067390
		0.6	−1.010570			0.6	−1.095500
		0.8	−0.907813			0.8	−1.120570
